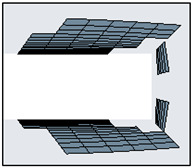# Parametric Study and Experimental Investigations of a Single Crank–Slotted Dual Lever Mechanism for MAV Flapping Actuation

**DOI:** 10.3390/biomimetics7040208

**Published:** 2022-11-21

**Authors:** Spoorthi Singh, Aravind Karthik Muralidharan, Jayakrishnan Radhakrishnan, Mohammad Zuber, Adi Azriff Basri, Norkhairunnisa Mazlan, Mohd Nizar Hamidon, Kamarul Arifin Ahmad

**Affiliations:** 1 Department of Aerospace Engineering, Faculty of Engineering, University Putra Malaysia, Serdang 43400, Selangor, Malaysia; 2 Department of Mechatronics Engineering, Manipal Institute of Technology, Manipal Academy of Higher Education (MAHE), Manipal 576104, India; 3 Department of Aeronautical & Automobile Engineering, Manipal Institute of Technology, Manipal Academy of Higher Education, Manipal 576104, India; 4 Institute of Advanced Technology (ITMA), University Putra Malaysia, Serdang 43400, Selangor, Malaysia; 5 Aerospace Malaysia Research Center (AMRC), Faculty of Engineering, University Putra Malaysia, Serdang 43400, Selangor, Malaysia

**Keywords:** bio-inspired, slotted lever mechanism, crank-slider mechanism, flapping flight, flapping wing micro-aerial vehicle

## Abstract

Insect RoboFlyers are interesting and active focuses of study but producing high-quality flapping robots that replicate insect flight is challenging., due to the dual requirement of both a sophisticated transmission mechanism with light weight and minimal intervening connections. This innovative mechanism was created to address the need for a producible structure that is small in size, small in mass, and has reduced design linkages. The proposed Single Crank-Slotted Dual Lever (SC-SDL) mechanism transforms rotational motion into specific angular motion at different velocities for each of its two strokes, i.e., the forward stroke and the return stroke. The discovery of a lag between the left and right lever motions in our design mechanism-I leads us to the conclusion that the flapping is asymmetric. To eliminate the position lag, the design has been altered, and a new design mechanism-II has been developed. Comparative kinematic analysis of both design systems is performed using simulations. Two-dimensional analysis of the base ornithopter configuration using ANSYS FLUENT yielded deeper insights regarding the influence of varying flapping frequency on critical flow metrics regarding adequate lift and thrust. For a flapping frequency of 24 Hz, adequate lift generation was achieved with minimal flow disturbances and wake interactions. Averaged dual wing estimations were made as part of the CFD study, which showed similar agreements. To validate the estimations, experimental tests were performed over the design mechanism-II configuration.

## 1. Introduction

A great deal of interest has been devoted to the design of bioinspired FW drones by the defense advanced research projects agency (DARPA) [1]; specifically, the science of dynamics and mechanisms, from the program initiated regarding aerial vehicles. Due to the outstanding flight strategies employed by insects and birds, numerous scientists have found inspiration in biological flight systems for the development of effective and suitable man-made flapping drones. In projects such as Microbat, KUBeetle, and Nano Hummingbird, in which lateral wing flapping is the core concern, numerous researchers have adopted a biomimetic design strategy. In comparison to rotating and static wing concepts, the flappable-wing technology provides the more sustainable technique. It could be since its force is delivered across a larger chord, swinging out of a boundary of minimum thrusts that elevates each side of the flapping position to a significant level (i.e., higher than in mid-stroke). Another outstanding feature of these flapping configurations is their layout as a versatile and effective oscillation systems with adequate flexibilities. The fluttering techniques require regulation across the wing by ensuring the control of both the wings’ activity, such as ceasing or changing of plane direction per stroke. The kinetic energy of the wing could be stored on the thorax walls, allowing it to be exploited there as conclusion per stroke to improve lift. The adaptability of flaps is a crucial element of flying movement that distributes aerodynamic forces efficiently [2,3,4].

The amazing feature of the flapping structures is that they are meant to be versatile, compact, and dynamic flapping mechanisms. Previous research has indicated that the duration of forward and return strokes of flapping during flight may not always be symmetrical. Alexander et al. [5,6] demonstrated that the downward flapping time of some dragonflies can be up to 2.37 times greater than the flapping duration of the upstroke. However, the time portion of artificial FWAV up and down stroke ratios is almost the same, hence, mechanisms of actuation providing symmetric flapping movements will always be preferred. Hence, yaw moments can be generated by utilizing the speed difference between the up- and downstrokes, even though the symmetrical flapping motions generates the major thrust force [7]. Sometimes, due to their symmetrically flapping wing movements, many modest motorized FWMAVs that can perform vertical liftoff are unable to achieve regulated hover. They also occasionally rely on their systems’ passive stabilization via the additional dampers’ influence on their flapping motions [8].

Author Hassanalian’s Thunder I [9,10] prototype integrates the variety configuration with the crank system design, with the exception that guiding bars are used rather than sliders. To produce a symmetric flapping motion at both wings, a six-bar mechanism with 14 connecting links across hinges was employed, yielding better lift. The insect-mimicking KU Beetle-S [11] uses a four-bar mechanism across a pulley with a string design framework to generate a clap and fling pattern of movement at both wing stroke variations, leading to greater lift. The higher stroke amplitude of FW and the additional payloads contributed to delivering greater lift and maximum flight duration. A small-bird type crank-rocker system [12,13,14] is structured with short compliant components. The main and secondary spars of the wing will deflect as a consequence of the oscillations, amending the camber with mid-chord velocities and thus influencing the lift and thrust forces. The primary spar’s compliance adjusts the observable wing area along and perpendicular to the flapping axis, ensuring stability at high harmonic distortion. The variation in overall thrust due to improved wing compliance is observed. The Saturn design model [15] utilizes a revolving crankshaft mechanism with a string-actuated two-linkage mechanism driven by an electric motor, in which each string controls the movements of the respective pulley system at its wing hinge. The oscillation/stroke movement is repetitive, but compared to the linkage’s actuated designs, the amplitude of the tip velocities is greatly reduced. Due to the design technique and wing pitch mobility, including spin and swirl dispersion, it was feasible to manage the pitch, roll, and yaw axes [16,17]. The design of the rotorcraft’s swashplate assisted to deliver control signals to actuation devices with a significantly narrower bandwidth than that of the oscillation frequency. As a result of the exceptional wing modeling approaches, the effect of flapping movement found to be the most effective throughout was wing membrane camber reversal. The contribution of flapping actuation and wing alignment in generating lift can be seen clearly in the above prototype design mechanisms. Due to the difficulty in designing and manufacturing less weight, higher efficiency, and compact-size FWMAVs, an effective design process is critical for achieving a better outcome [18,19]. By conducting a literature survey on flapping wing actuation systems, the authors of this paper discovered that the slotted-lever actuated similar to quick-return mechanism has not yet been adopted in flapping applications [20,21].

A comprehensive review of the literature on various configurations shed great deal of light on the several critical parameters influencing the design of FWMAV. While efficiency and design objective remain the primary goal of prototypes, the most critical factor of manufacture complexity and ease is often less considered. The above-specified design prototypes are of complex mechanical configurations, which also adds to the net weight and wing span of the model. In our study, for the basic expected aerodynamic characteristics, a compact, simple, and more robust fast backward stroke mechanism is modelled, which tackles the disadvantages of previous configurations, both in terms of weight and manufacturing complexity. Current research trends for developing lightweight flapping prototypes generally choose material actuations such as piezo, dielectric, electrostatic, etc. However, a motor-actuated model is seldom explored. A good comparison for theoretically gauging the effectiveness of the proposed mechanism is juxtaposition with Robobee [22,23], an ultralight insect-mimicking flapping-wing vehicle with a weight of <2 g, but achieved using a material actuation instead of motor actuation, which has its merits [20,24,25]. In addition to slashing down the complexity of the model whilst using a fast backward stroke mechanism, the proposed mechanism also bolsters the applicability of motor actuation in ultralight, insect-mimicking flapping-wing vehicles and thus acting as a proof of concept.

Computational understanding of the dynamics of animal flight to explain the physics of these creatures is the key to developing a flapping-wing aircraft. For a successful micro air vehicle design, scrutinization is to be laid on three critical factors–level of control, agility, and the associated thrust-power distribution rendered by the pattern-specific flapping motions of these creatures [26,27]. A comprehensive investigation of its dynamics and critical aerodynamic flow parameters aids in the juxtaposition of its aerial kinematics against other proposed research models of the MAV, whilst ensuring its validation as a prospective novel build. This study aimed to convey the influence of flapping frequencies, pitching angles, and diverse combinations of wing designs on static thrust and generated power from the flapping motion. A single-crank and slotted dual-lever mechanism for actuating flaps in the proposed MAV wings here in this paper. The ratio of upward and downward flaps is different due to its quick return characteristics. As a result, this mechanism has been validated through experimentation and numerical simulations of flight characteristics. Physical parameters include flapping intensity, upward/downward flapping angles, angular velocity, angular acceleration, and flapping frequency of the system design, analyzed through simulations. Two flapping actuation mechanism prototype designs are built and compared to choose which is best among them, using real-time observations and experimentation strategies.

## 2. Concept of Design Mechanism-I

**Figure 1:**
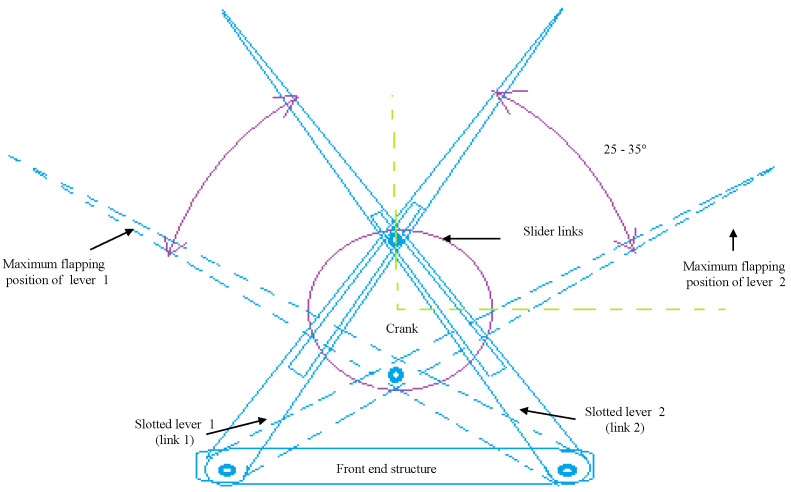
SC–SDL design mechanism—I.

Generally, a slider actuated crank connection is a four-bar linkage system, made up of three revolute joints and one prismatic (or sliding) joint, used commonly in flapping-wing aerial vehicle application., where the rotation of the crank drives the specific angular movement, or the slider is creating flaps of upward and downward strokes at the lever in any FWMAV. Slider cranks are classified into two main types: in-line and offset. The slider on an in-line arrangement is placed such that the line of transit of the slider’s hinge joint moves according to the source joint of the crank, i.e., as the crank spins, this causes a corresponding symmetric slider motion in an up-and-down pattern. While in the slider on an offset arrangement, the line of transit of the slider’s hinge joint does not pass via the source pivot of the crank, causing the slider motion to be asymmetric. However, it moves more quickly on its returning path than on the forward path of its motion. This is referred to as a quick-return mechanism. Using the slider crank mechanism as a reference model, a novel design known as the Single Crank-Slotted Dual Lever (SC-SDL) mechanism is being created, as shown in Figure 1. The basic design for converting rotary motion to specific angular motion in this case is the arrangement of two quick-return mechanisms in an X fashion. This unique arrangement of the quick-return mechanism is referred to here as the single-crank and slotted dual lever mechanism, as shown in Figure 1.

**Figure 2:**
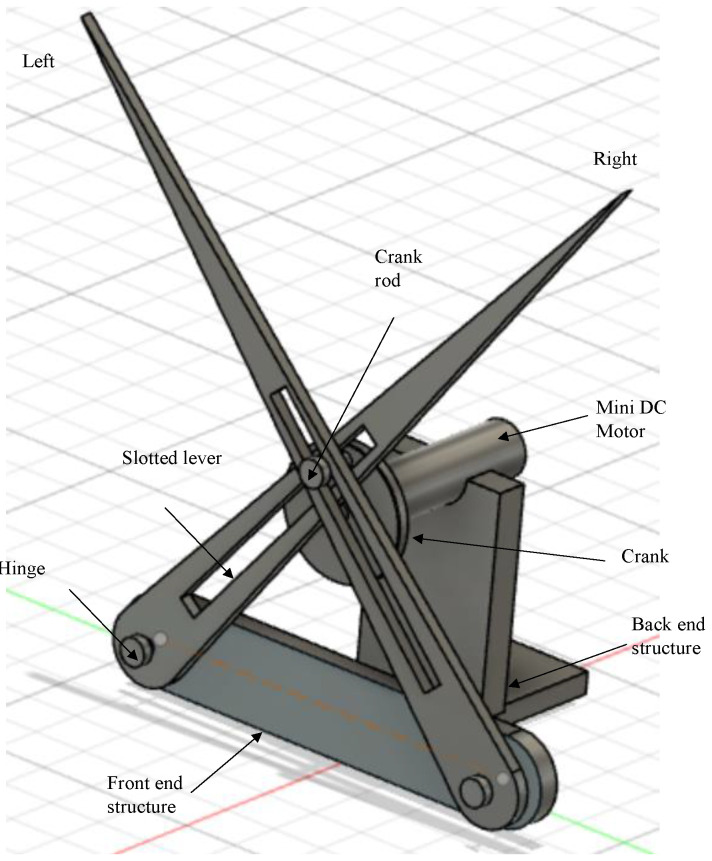
CAD model of SC-SDL Design-I motor driven Single-Crank-Dual-Slotted Lever Mechanism for flapping actuation.

**Figure 3:**
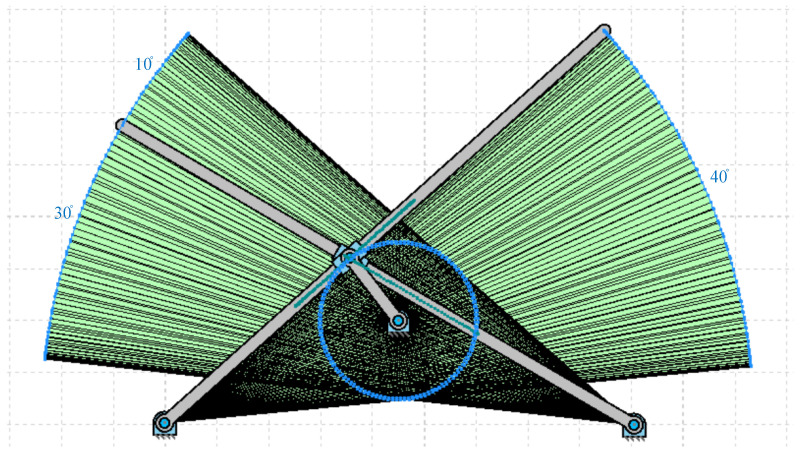
Workspace of the modeled SC-SDL design mechanism-I.

The quick-return mechanism is composed of an arm connected to a disc that revolves at a consistent speed. Based on the movement of the crank, the arm slides along the slotted lever, causing the lever to swing forward or to return the stroke angle pattern. However, at the other side, the angle of forward motion of the lever is greater than the angle of reverse motion. Consequently, the time required for the lever’s forward stroke exceeds the time required for each lever return stroke. Since the return motion of the lever is quicker than its forward motion, this mechanism is known as the quick-return mechanism. A single-crank dual slotted lever mechanism is being utilized to generate a repeated particular angle of flapping motion driven by a crank rotation. It performs a similar function as a slider-crank mechanism or a quick return concept, and it is particularly helpful when the necessary stroke of the actuated flapping angle is less in comparison to the dimensions of the driving crank system. As the motor rotates, the crank connected to it begins to rotate in the same direction, causing the crank to drive both slotted levers in the opposite direction. The lever will move in a specific angular motion, i.e., pitch action in the opposite direction, creating a 25°–35° angle of motion. By connecting the wings at the tip of the slotted lever, the flapping motion of the lever could be used to generate lift in FWMAV applications. One full rotation of the piston causes the slotted lever to create two flaps, i.e., upward and downward strokes. 3D modelling of the mechanism is designed in CAD and its simulations are performed for the lever motion analysis as in Figure 2 and Figure 3 respectively.

### 2.1. Parts Description of the Design Mechanism-I

The basic components of the flapping actuation mechanism design-I are best described using Figure 2, with its CAD modeling in Fusion 360 software. It consists of the following components:

1. Base–serves as a support for the front- and back-end structure frames.
2. Front end structure–a frame that supports two lever slots that are connected across it.
3. Backend structure–consists of a triangular-shaped supporting frame that holds the motor.
4. Motor–a micro-DC motor for rotation that is linked to a crank.
5. Crank–a rotary gear-like structure with no teeth and a rod-like projection on one side.
6. Slotted lever–two levers with rectangular slots in the middle that connect to the crank in an X pattern.

### 2.2. Simulation Results of Design Mechanism-I

In this section, the operation of the flapping actuation mechanism with the required linkages and its kinematic analysis is reported using the GIM^®^ software, which is a registered software of the Department of Mechanical Engineering, UPV/EHU [28]. The design mechanism-I with a single crank and dual slotted levers is modelled in the GIM^®^ software, and its generated work space is presented in Figure 3 below. The angle formed at both the levers is the same, i.e., 40 degrees. However, the phase lag between the left and right wing is 10 degrees, as shown in Figure 3, i.e., when the right lever tip is at 40 degrees, the left-wing tip is at 30 degrees.

**Figure 4:**
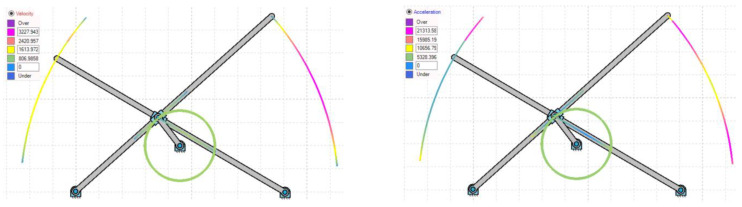
Color representation of variations in velocity and acceleration at both lever paths of motion.

**Figure 5:**
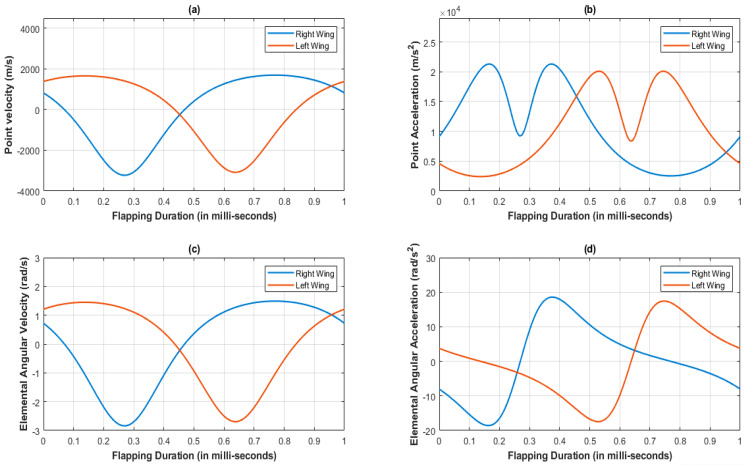
Kinematic analysis at lever tip of the SC-SDL design mechanism-I; (**a**) Point velocity vs. Flapping Duration; (**b**) Point Acceleration vs. Flapping Duration; (**c**) Elemental Angular velocity vs. Flapping Duration; (**d**) Elemental Angular Acceleration vs. Flapping Duration.

Figure 4 represents the variations of under- and overintensities of velocity and acceleration during both lever motions, clearly indicated by color at each instance. The asymmetric slider motion is evident from the colored simulated output of velocity and acceleration in Figure 4. The lag between both lever flapping motions is due to the offset type of a crank slider mechanism’s natural characteristic and the X pattern of alignment between the levers. Figure 5a,b indicates the results of point velocity and acceleration analyses performed with the GIM^®^ software.

The velocity at the left and right lever tip points are completely opposite to each other; that is why there is a phase difference and speed difference between both lever motions. Additionally, the variation in angular velocity and acceleration with respect to left and right slotted lever elements is observed at Figure 5c,d.

The above proposed design mechanism-I can generate flaps at its lever, but its velocity and acceleration vary greatly. This is due to structural design constraints, i.e., the time of the flapping of both levers is not the same but varying. As a result, the above mechanism design-I is not creating symmetric flapping, hence it is incompatible with flapping wing MAV applications. However, by making minor changes to the design mechanism-I, it has yielded a revised design model known as mechanism design-II, as shown in Figures, where the revised design mechanism-II can minimize the phase lag between the flapping coordination of both wing motion paths with respect to time.

## 3. Crank and Lever Transmission of Design Mechanism-II (Revised Design)

**Figure 6:**
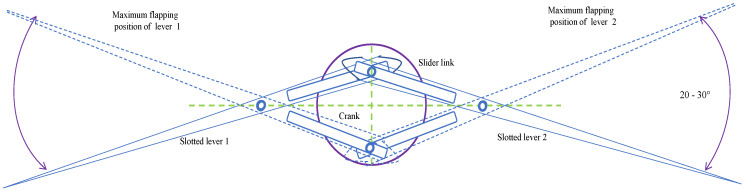
SC-SDL design Mechanism-II.

**Figure 7:**
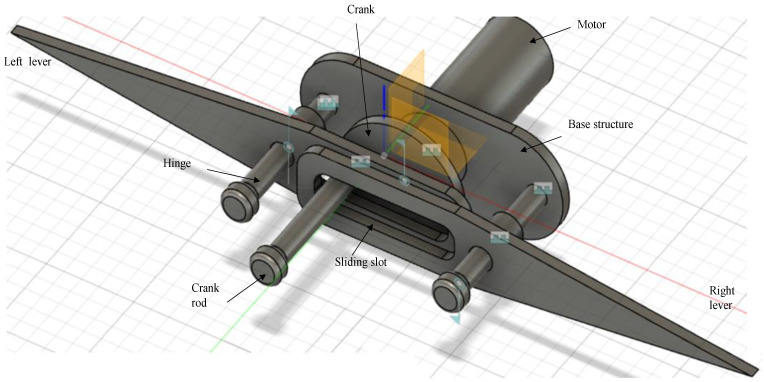
CAD model of SC-SDL Design-II, a motor-driven single-crank dual slotted lever mechanism for flapping actuation.

In this revised design mechanism-II, both the hinges and the motor shaft with the crank are aligned to the origin of the horizontal plane. In comparison to the design mechanism-I, the lever in this case is also extended beyond the hinge connection and is regarded as a flapping lever tip. As illustrated in Figure 6, both levers are placed opposite each other. The front-end and back-end structures are avoided in this revised mechanism to reduce the overall design weight. The base structure is used here on the motor’s top edge to provide support for both hinges. As in the first design, a single motor drives the crank, and the crank’s rotation is converted to specific angular motion/flaps by a sliding arrangement at one end of the lever via its slots. The angle of motion produced at its hinge connection will be almost 20–30 degrees, which is slightly less when compared to design-I flapping angle. Figure 7 depicts the CAD modelling of the revised design mechanism-II.

### 3.1. Parts Description of the Design Mechanism-II

Figure 7 best illustrates the basic components of the flapping actuation mechanism design-II, which was CAD-modelled using Fusion 360 software.

1. Base structure–serves as a support for the hinges and levers, fitted on the motor top edge.
2. Hinges–a bolt-and-nut idea structure that stands on the base structure to provide support for two levers along its holes that are coupled.
3. Slotted lever–two levers with rectangular curved slots on one edge (that surround the crank rod by stacking on each other) and a sharp tip on the opposite edge of the lever (where the wings will be attached)
4. Motor–a miniature DC motor for driving the crank, which indirectly drives the slotted levers.
5. Crank–a circular framework with no teeth and a rod-like extension on one side.

### 3.2. Simulation Results of Design Mechanism-II

**Figure 8:**
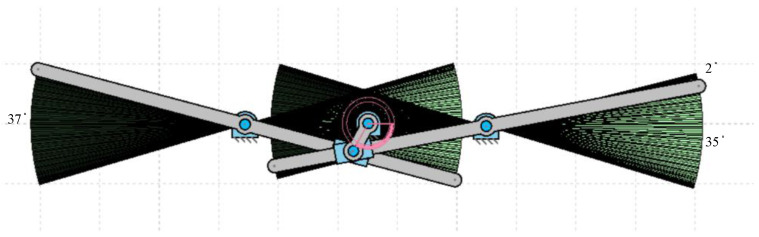
Workspace of the modeled SC-SDL design mechanism-II.

The flapping motion of the slotted lever design mechanism-II could be used to generate lift FWMAV applications by connecting the wings at its lever tip. With one full rotation of the piston, the slotted lever produces two flaps, i.e., upward and downward strokes. The simulation modeling of the lever motion analysis with workspace is shown in Figure 8. The angle generated by both levers is 37 degrees. Figure 3 illustrates that the phase lag between the left and right wing is 2 degrees, thus while the right lever tip is at 37 degrees, the left lever tip is at 35 degrees.

**Figure 9:**
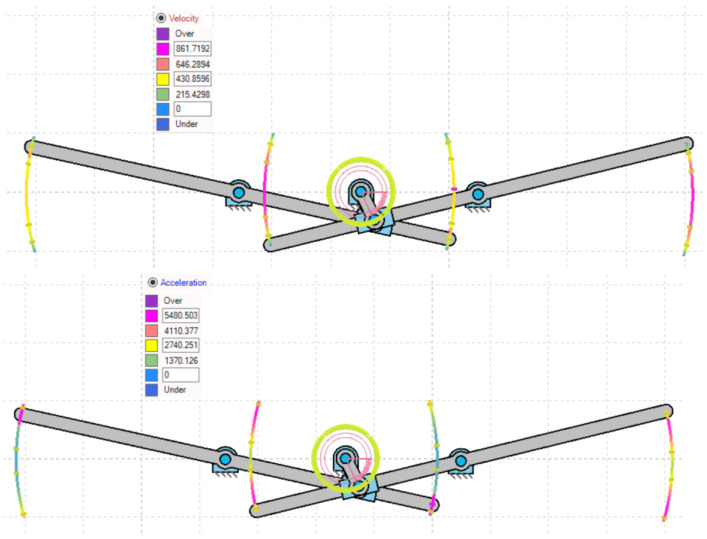
Color representation of variations in velocity and acceleration at both lever paths of motion.

Figure 9 shows the variations in the speed and acceleration levels below and above, with a clear color indication in each case. From the colored simulated velocity output and acceleration in Figure 9, the asymmetric flapping is noticeable. Since both levers are operated by a single crank, the velocity distribution along their paths is distinct. However, due to the reconfigured orientation of the lever and crank connections, the shortfall between the lever flapping is reduced in comparison to the first design.

**Figure 10:**
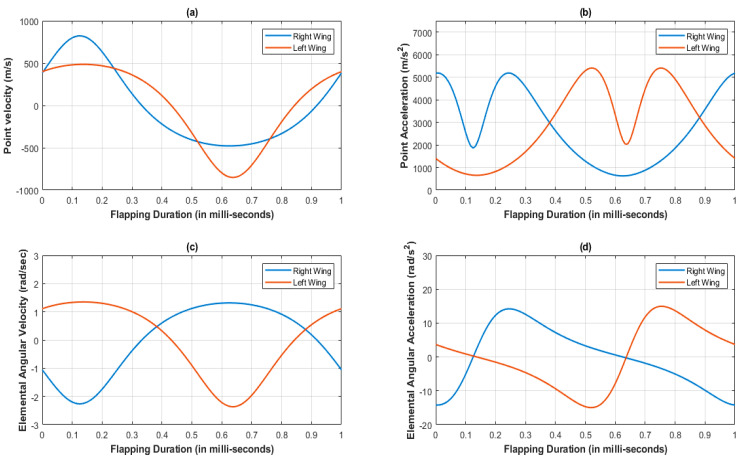
Kinematic analysis at the lever tip of SC-SDL design Mechanism-I; (**a**) Point velocity vs. flapping duration; (**b**) Point acceleration vs. flapping duration; (**c**) Elemental angular velocity vs. flapping duration; (**d**) Elemental angular acceleration vs. flapping duration.

Figure 10a,b illustrates the summary of the GIM^®^ software point-velocity analysis, demonstrating that at the left lever element there is a small decrease in speed when compared with the right lever element. Figure 10a,b also illustrates the decreased point velocity ratio between the left and right lever tips. However, the acceleration patterns observed in design mechanisms I and II are identical. In addition, the angular velocity and acceleration of the lever element are comparable in designs mechanism I and II as in Figure 10c,d. In design mechanism-I the phase lag is 10 degrees, while in design mechanism-II the phase lag is 2 degrees. However, the phase delay between the wing’s movements is minimized in the revised design of mechanism-II, so this design could be suited for FWMAV applications. Hence, the authors of this paper attempted a parametric study for its motional analysis, which is elaborated upon in the further sections.

## 4. Computational Fluid Dynamic Study and Results of Design Mechanism-II

A comprehensive investigation of the dynamics and critical aerodynamic flow parameters for design mechanism- II aids in the juxtaposition of its aerial kinematics against those of other proposed research models of the MAV whilst ensuring its validation as a prospective novel build. Mathematical formulations in the early investigations of a flapping wing and propellers leveraged the capabilities of steady aerodynamic theory to flapping biomimetics. To mathematically simplify the dynamics of flapping wings, Osborn discovered that at a predetermined moment, flap positioning with a certain instantaneous speed and degree of approach provides the expected amount of immediate thrust and wing lift [29]. This kind of estimation involves averaging the aerodynamic forces over the planform of the flexible wing [30], per stroke, where the resolved instantaneous horizontal and vertical elements of the overall force are contrasted with the thrust and weight forces respectively to arrive at the averaged versions of the drag and the lift forces. This technique is known as the quasi-steady-aerodynamic concept of flapping flight. Despite its successful implementation and validation in intermediate subsonic regimes for fast horizontal motion, its applicability to lower subsonic motions (which is the regime of operation for several micro-aerial vehicles) remained unexplored with little to no comprehensive study to corroborate the claim. In another noteworthy research by Singh and Chopra, the performance of flapping wings with articulated pitching was comprehensively studied at controlled conditions of external wind dynamics. This study aimed to convey the influence of flapping frequencies, pitching angles, and diverse combinations of wing designs over static thrust and generated power from the flapping motions. Time marching stroke averaged analytical schemes were utilized in this particular study to understand the important aspects of flapping aeromechanics in addition to lift and drag estimation, such as dihedral offset, stiffness of wing material, constraint of wing edge motion, variation in wing size, alteration of stroke plane angle from horizontal to vertical, application of momentum theory for oscillating wings, in designing FWMAV. Liu et al. presented a fast and efficient method [31,32] based on Delaunay graph mapping for computationally analyzing the flexible wing dynamics in a three-dimensional setup. In addition, a variety of computational schemes were tested to evaluate the kinematics design process for controlling the insects’/birds’ motion based on the variations with respect to the count of wings, their layout, and wing design characteristics.

For the present proposed design, a geometrically simplified 2D cross-sectional model was considered. Investigating the dynamics of the proposed design mechanism-II, this simulation simplification is well-justified, as the extended arm initiates a fast-return “rigid body” motion without any pivots to account for the flexibility of the spar of the wing. To model the stream across the adaptable wing, a mechanism must be developed to modify the volume mesh at each interval. Three-dimensional analysis with higher order solvers accounting for greater degrees of freedom can effectively capture the near tip wake, fluid-turbulence interactions, and associated stagnation and transition effects. However, the flexible sheath over the rigid spar in the 3D study will be subjected to noticeable and computationally significant deformation, which poses immense challenges in domain discretization and hence makes the convergence of turbulent kinetic energy (k) and specific dissipation rate (omega) challenging. Presently, there are two ways to solve the mesh deformation problem: the spring analogy method and the elastic body analogy method. The spring analogy model utilizes the method of connecting each node in the neighboring cells by springs, within which the governing equations for the fluid flow and the equations of motion for the mesh deformation are integrated in time to compute the reformed mesh cell position. This method works well for small motions but leads to high skewness and eventually grid invalidity for larger motions. Contrarily, in the elastic body analogy, the entire grid itself is considered as an elastic body where for the entire domain, the fundamental partial-differential equations are resolved. While it does resolve the issues of its counterpart, it takes its toll in terms of computation, and for a few cases in terms of accuracy (specifically for unsteady and highly turbulent flow problems), owing to the spatial discretization schemes employed. As the scope of the current study is to validate the MAV design with computational study, while restricting the nuances, a nondeforming, transient 2D study of a frequency-controlled flapping wing cross-section is performed. Kinematically analyzed heave and sink motions of the quick-return flapping wing from the analysis data mentioned in Section A could be imposed in the CFD methodology to assign necessary initial conditions for computing the aerodynamic loads. Acquired data post-proper grid independence verification can be probed further to increase the study of the aerodynamic systems of flexible wing-flapping [33].

### 4.1. Geometry Simplification

In design mechanism-II, the pitching of rigid wings is realized with the help of an articulated pitching mechanism. The flapping motion is actuated by a leading-edge spar over which a flexible membrane with a 1.2-degree washout between the root and the tip is attached. A 0.12 rad dihedral angle is given for the entire flapping wing setup to better account for effective thrust-propulsion distribution, as suggested by Roget et al. [34,35,36]. A two-dimensional wing projection in the XY plane as a flat plate with dimensions of 37 mm length with net assumed thickness of 5 mm was modelled within the Space claim CAD modelling tool. For CFD simulation, the tail is not modelled, and only the pair of wings is considered, as design mechanism-II focuses on the wing dynamics.

The dynamic action of the flapping wing, subjected to varying angles of attack amplitudes, will be the victim of turbulence-induced flow separations and flow obstructing wake, as well as dead zone formation. To better capture the effects of turbulence and to support steady flow development before interaction with the wing projection, a computational domain long enough was created, specifically after the trailing edge was modelled. Considering H as the reference length, which in most cases could be made synonymous to the chord length of the wing projection, the computational domain ahead of the leading edge was considered as 10H and the domain to the rear as 15H.

**Figure 11:**
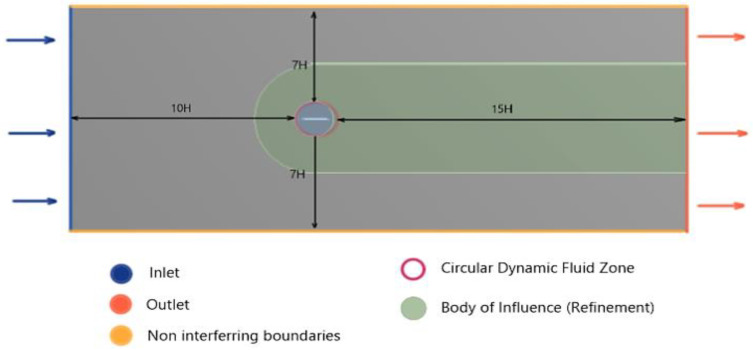
Computational domain for the analysis of the flapping design mechanism-II.

Boundary conditions playing a significant role in the modelling of the computational domain, as well as careful modelling of the top and the bottom models, are to be considered. Specifically for flows with varying degrees of angles of attack, flow rebound effects will be more pronounced if the top and bottom bounding surfaces are modelled as walls. To reduce these flow rebounding effects, the top and bottom walls are to be modelled far apart, for example 12H–15H. However, as they immensely increase the computational time, the top and bottom boundaries are modelled as symmetric zones with 7H clearance from the flat plate zone, each. Finally, to account for the dynamic motion of the flat plate within the grid and to locally refine the trailing edge zone, a circle with a 42 mm diameter and C grid-type bodies of arbitrary dimensions were created. These generated zones will aid in defining bodies of influence. A graphical depiction of the created computational domain is illustrated in Figure 11.

### 4.2. Domain Discretization

The scientific literature and various studies corroborate the information that the element size has a direct influence on the accuracy of the computational results. This greatly depends on the kind of algorithm adopted to decompose the domain. While a highly fine mesh yields reliable numerical predictions with minimal error in contrast to its coarser counterpart, a finer mesh takes a toll on the computational cost, time, and effort.

Solver-specific tried and tested grid generation methods were used to discretize the entire domain and solved using Incompressible Reynolds Averaged Navier-Stokes’s equations (RANS). Basically, RANS equations were constructed by the averaging over time of the N.S. Equations. By simulating Reynolds stresses, the impact of disturbance can be approximated. Probing into the literature, conventionally three types of grids are commonly incorporated, namely, structured, block-structured, and unstructured grids. Original mathematical formulations for finite element, finite difference, and finite volume are based on the consideration that the entire domain is divided uniformly into square grids. The underlying flux transfer and node parameters preconditioning is also based on this assumption. So, while using structured grids seems like an ideal choice, considering the merits of cell order and efficient flow field computation, it is a challenging fit for complex geometries or dynamic meshes. A flat plate is not a complex geometry, but the dynamics of the flat plate for the parametric flapping frequency study is indeed the prime focus, for which unstructured grid offers better fit and mesh quality over its structured counterpart [37].

**Figure 12:**
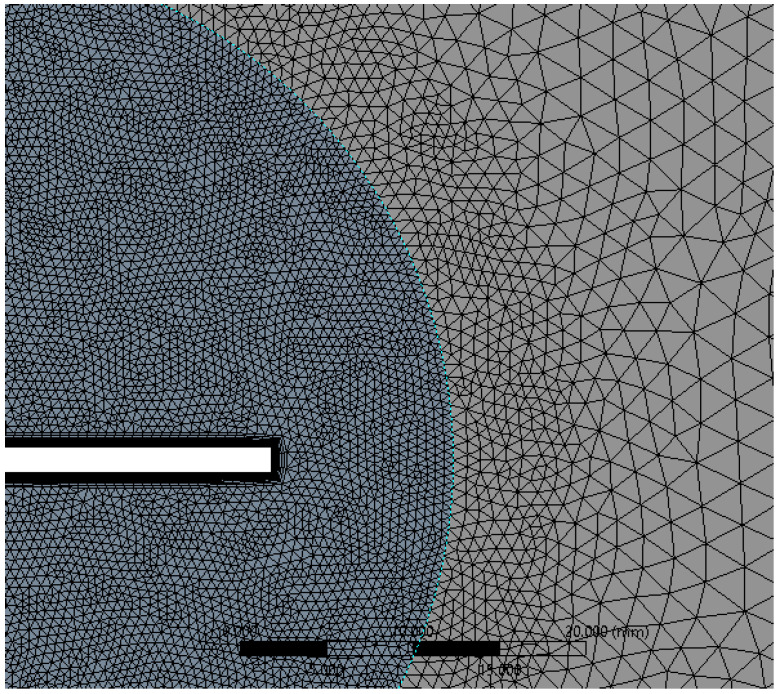
Local refinement within the dynamic and static fluid zones for uniform residual convergence.

The simulation is set up for a parametric study with four flapping frequencies from 20 Hz to 45 Hz. The entire computational domain was split into triangulated grids with a uniform grid element size of 10 mm. This grid size is gradually clustered towards the circular zone enclosing the flat plate, to account for better capture of the near wall effects as shown in Figure 12. The clustering towards the circular region was unbiased, with a linear element order and a curvature capture minimum size of the order of 0.1 mm. Mesh defeaturing, although toggled on, was not of prime concern here, due to the simplistic geometrical consideration devoid of higher-order curvatures.

**Figure 13:**
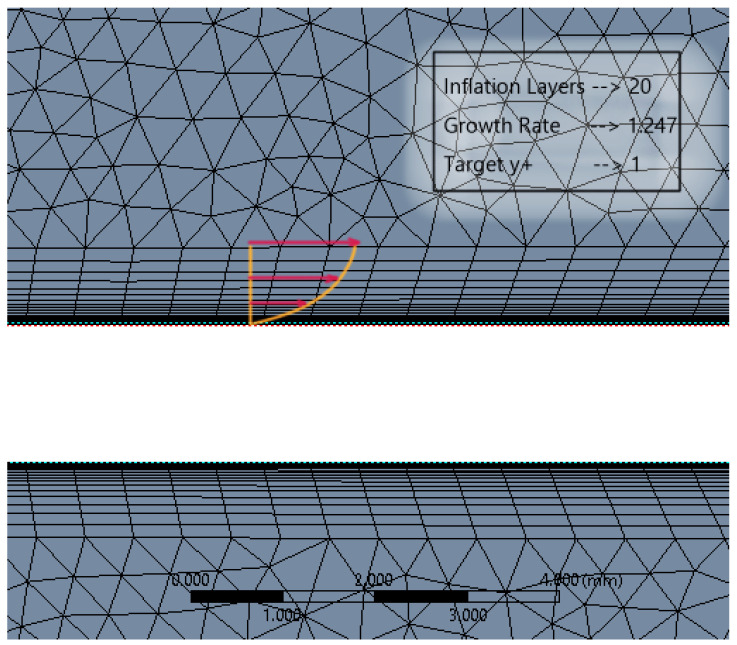
Boundary layer inflation zone in close proximity to the flat plate.

In accordance with the inflation layer mathematical requirement for the considered reference length, a 20-layer inflation layer over the surface of the flat plate with a growth rate of 1.247 was constructed (see Figure 13). This grid clustering near the surface is paramount to predicting the aerodynamic forces and the varying static pressure over the geometry. The boundary layer is a very minute zone within which the viscous effects of the fluid become more pronounced. This tugging action of the fluid has a direct influence over the drag estimation. Hence, inflation layer control is required to create thin elements that can capture the normal gradient with a minimal cell count.

(1) $$y^{+}=\frac{\rho y_{p}u_{T}}{\mu }$$

**Figure 14:**
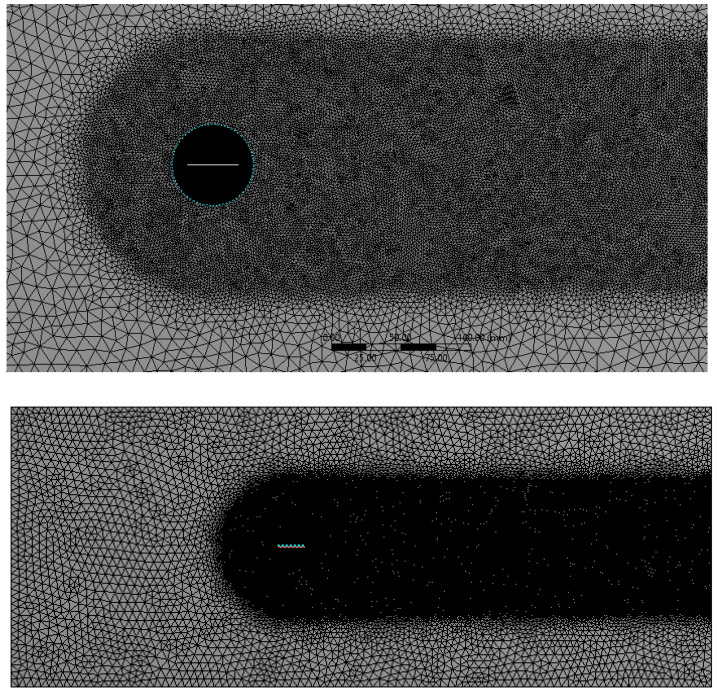
Local capsular discretization to capture the wake distribution of the separated flow.

As specified earlier, wake capturing is key to understanding the aggregate flow separation effect, which in turn also determines the thrust-lift distribution due to the flapping motion. Hence, a body of influence meshing criteria was given for the C-shaped body stretching till the end of the domain, as shown in Figure 14. Each element within this body was sized at 2 mm.

For such dynamic flapping motion, it is possible to implement CFD solver algorithms to mathematically morph the nodes and the grids to support the motion of the flat plate. ANSYS FLUENT supports a variety of mesh motion techniques to aid in transiently morphing the grids in accordance with the varying shape of the flat plate. In this regard, the circular region enclosing the flat plate was modelled as a flapping body with the aid of UDF scripting. Using the in-built CFD C++ code interpreter, the script was compiled and executed to induce flapping motion for the circular region. A prime focus was to attain the necessary motion while ensuring quality, efficiency, and robustness. With all these considerations, smoothing dynamic mesh motion was implemented and the entire domain was discretized (see Figure 14). Due to the dynamic skewing nature of the morphing mesh between the interacting interface of the circular zone (here, the internal region enclosing the flapping flat plate) and the outer zone, representing the fluid domain, the mesh orthogonal quality and the overall mesh quality becomes compromised at higher angles of attack (AOA) limits for the flapping mechanism. In such cases, it is suggested to prefer geometry motion instead of dynamic mesh motion. Owing to the lower AOA limits of operation and to the ease of case setup with dynamic mesh motion, the authors here have preferred this setup and obtained suitable mesh quality as summarized in Table 1.

### 4.3. Solver Setup

**Figure 15:**
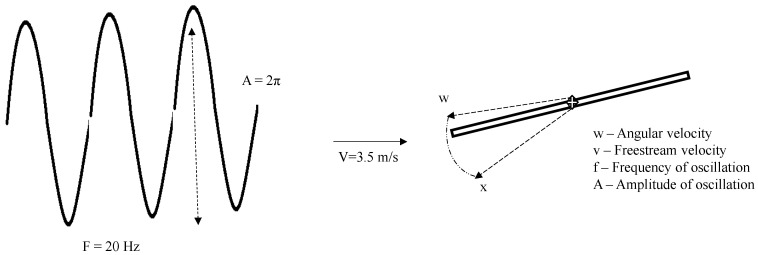
Flapping physics for the computational fluid dynamic ornithopter analysis.

The flapping movements are constrained to the stroke planes, in which two separate phases of pitching are considered, i.e., pronation (forward-backward) and supination (i.e., vice -versa). For the computation of turbulence in the inviscid flow domain, attributing to the low speed of operation, a pressure-based solver with iterative two-equation k-omega SST RANS turbulence and low Reynolds number correction was used. The objective is to study the transient effects of the flow, due to which the entire simulation was carried out as a function of time. Incoming wind velocity was along the +x direction, having a magnitude of 3.5 m/s operating at standard atmospheric conditions. The key to setting up the flutter motion is to add dynamic frame and mesh motion, which, for each of the interior and exterior interface zones, were modelled with UDF. Based on design mechanism-I experimentation results, the flapping frequency for the base design mechanism-II model was tested parametrically for 20 Hz, 30 Hz, and 40 Hz with amplitude of 2*PI, fixed based on the degree of motion of the mechanical spar (see Figure 15). To better understand the transient effects and infer the net thrust force generated along the axial direction, the simulation was run for a total of 43,500 iterations, time stepping in every 0.001 s to obtain the total simulation result for 1 s.

The evaluation of the inviscid fluxes is based on an upwind-biased flux-difference technique proposed by Roe [38,39] For time-accurate computations a second-order COUPLED scheme in time is used, along with Newton-type sub-iterations to remove factorization errors and restore formal second order accuracy. For spatial discretization, a linearly governed Least Square Cell Based scheme was deployed with second order iterative computation for pressure and third-order MUSCL method for other flow and turbulent parameters.

### 4.4. Inferences

Based on the simulation data acquired from the aerodynamic analysis of the simplified ornithopter 2D geometry, it could be inferred that the design mechanism-II offers a feasible means of operation from an aerodynamic standpoint. The flapping wing rendered a peak lift coefficient of 0.46 at its 0.2 s time of operation and a peak drag coefficient of 0.08. Transient Velocity contours were probed at five timestamps within its 1 s of operation to understand the dynamics of the flow upon its interaction with flapping flat plate geometry.

**Figure 16:**
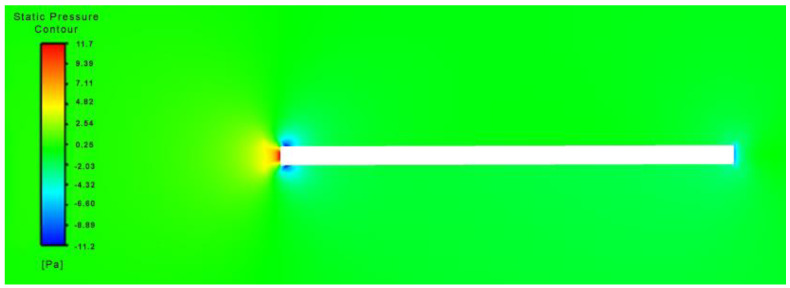
Static pressure distribution over flapping flat plate at t = 0.001.

During its initial state of operation, it was assumed that the flapping wing had no dihedral angle in relation to the body, and hence an angle of attack of 0 degrees was maintained. Incoming flow stagnates at the leading edge of the flapping wing’s spar with a symmetric pressure distribution and a dead high-pressure zone at the rear as depicted in Figure 16.

**Figure 17:**
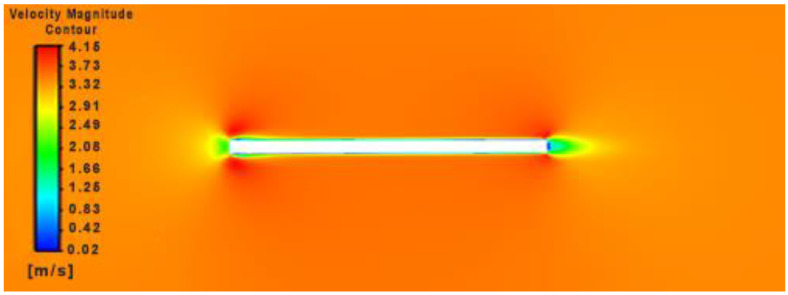
Velocity magnitude contour over flapping flat plate at t = 0.001.

Owing to the tugging effect of the near wall shear, upon a close encounter of the flow with the body and due to the gradual development plus external compression of the growth of the boundary layer by surrounding pressure, it can be noted that the incoming fluid takes a curvilinear trajectory (a characteristic of the subsonic fluid) and then tucks within a narrow layer near the body as shown in Figure 17.

**Figure 18:**
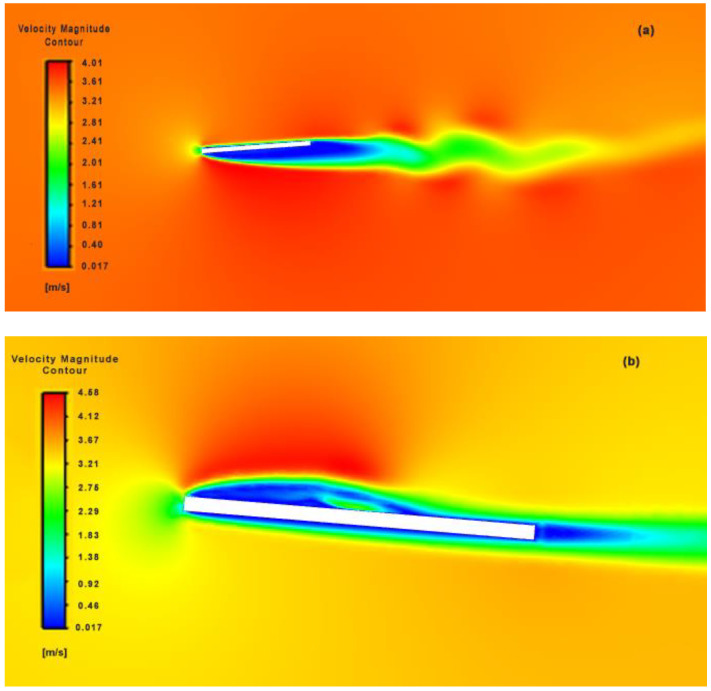
Symmetrical (**a**) downstroke and (**b**) upstroke velocity magnitude contours over flapping flat plate at 150th and 314th time frame respectively.

Comparing its 150th and 314th flutter velocity contours (see Figure 18), separation produced on the upstroke and downstroke is asymmetric, which in turn generates a larger flow separation on the lower side of the flapping body in contrast to its upper side. This could be attributed to the center of control where the flapping motion is initiated in our current design mechanism-II model and due to the adoption of a bioinspired quick-return control mechanism. The resultant vortices created by the wing are pushed towards the rear, which creates a net horizontal momentum leading to thrust production.

**Figure 19:**
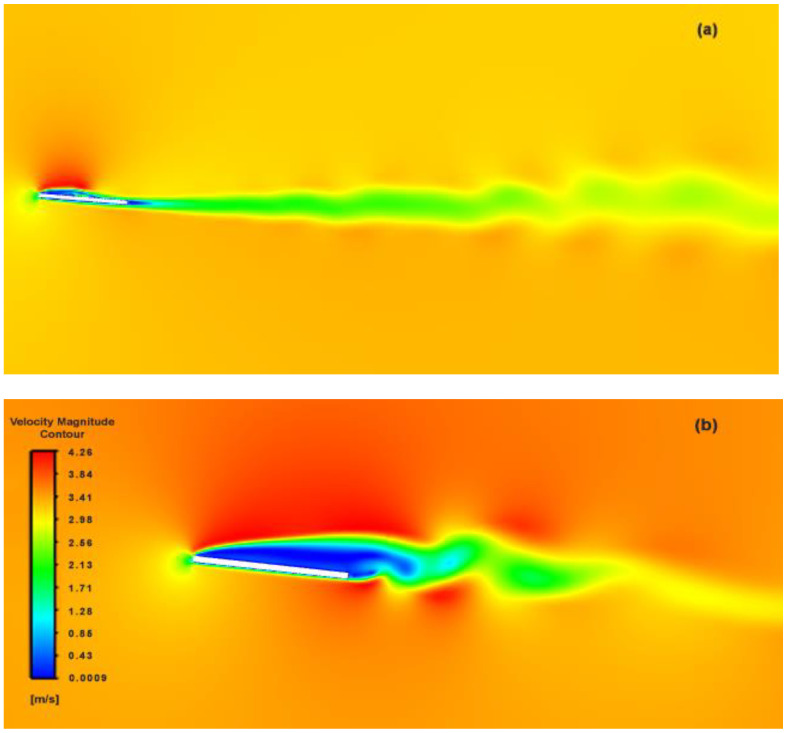
Upheave wake-induced flow separation over flat plate; (**a**) wake distribution, (**b**) magnified view.

As the flapping progressed, the wake disturbance from previous strokes had obvious and conspicuous impacts on the flow adherence of upcoming strokes (see Figure 19), which resulted in larger wake formation and premature flow separation. These vortices were found to form along the leading edge with gradual motion towards 53% of the flat plate geometry, at which point the flow became highly unstable and detached from the plate’s surface. This did take its toll on the net thrust produced; however, the impact on the aggregate thrust drop due to the flow separation had little impact.

It appeared that the thrust force is higher for a higher dihedral angle. One common observation was that a higher dihedral angle extended the zone of flapping, rendering higher thrust force, but with an obvious bottleneck of limitation in the stall angle. However, the nonuniform pressure distribution with dynamic flapping had a great merit over its fixed-wing counterpart, as flapping wings do not exhibit a typical, abrupt stall seen with the fixed wings. This offers a greater flexibility and window for control.

**Figure 20:**
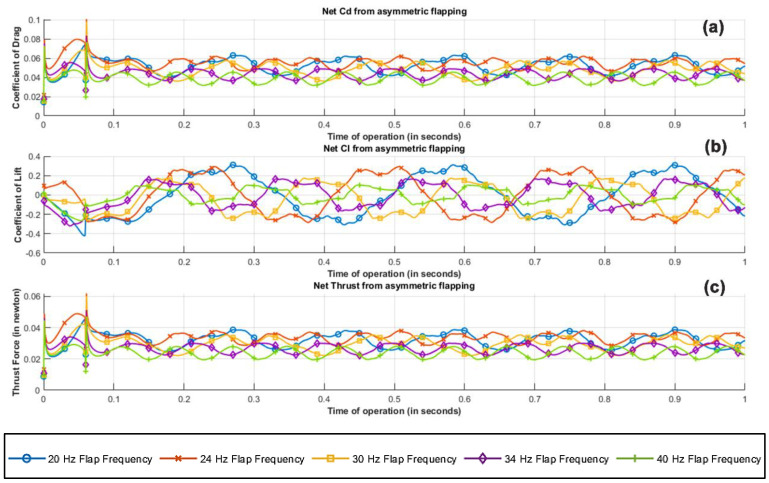
Net flow field metrics of asymmetric flapping plate configuration; (**a**) coefficient of drag, (**b**) coefficient of lift, (**c**) thrust force along the positive x axis.

Graphical interpretation of results from the transient CFD parametric study rendered key insights about the performance of the figure of the eight-flapping-wing mechanism. Based on the simulation results from 1 s of operation for frequencies ranging from 20 Hz to 40 Hz, fluid dynamic characteristics, namely, coefficient of lift, coefficient of drag and effective thrust for both individual flapping plate and combined flapping wing mechanism were analyzed. Critical analysis of the performance from Figure 20 indicates that there is a gradual hike from 20 Hz to 24 Hz and a drastic dip from 24 Hz to 40 Hz in the effective lift and drag characteristics of the flapping flat plate. While a higher flapping frequency guarantees effective improvement in the lift obtained due to the structure operating and turning a larger mass flux of incoming air, the induced turbulence caused by the flat plate at a higher frequency of operation outweighs the net benefit. In addition, as the wing at higher flapping frequencies is on the lower subsonic spectrum, the flow is prewarned about the presence of the body upstream, which after certain flaps encounters an already disturbed flow leading to additional induced drag. The current test body with its flapping mechanism experiences a delay of 60 micro seconds between the flaps of the right and left wing. To simulate and interpret realistic flow conditions over the test body, the time delay between the flaps was accounted, and by mathematically superimposing the phase difference, a weighted average was computed.

Let ‘t’ be a column matrix of dimension mx1 denoting the simulation time of the ornithopter study from 0 to 1 s in steps of 0.001 s. At 60 ms, the corresponding index of the 0.06 s time stamp is calculated as

0.06 = 0 + (n − 1) ∗ 0.001(2)

(3) $$n=\frac{0.06}{0.001}+1=61$$

If $C_{L\left(R\right)}$ denotes the lift coefficient matrix of the right wing, which has the same matrix dimension as that of ‘t’ matrix (i.e., mx1), then the $C_{L\left(L\right)}$ matrix (i.e., the lift coefficient matrix for the left wing) is represented as

(4) $$C_{L\left(L\right)}=\left[\left[C_{L\left(R\right)}{\left[m-61:m\right]}_{61x1}\right];\;{\left[C_{L\left(R\right)}\left[61:m-61\right]\;\right]}_{m-61x1}\right]$$

i.e., due to a shift by 60 ms, the 1st $C_{L\left(L\right)}$ value will be equivalent to the 61st $C_{L\left(R\right)}\;$ value. The last 60 values of $C_{L\left(R\right)}$ will be the starting logged coefficients for the left wing.

**Figure 21:**
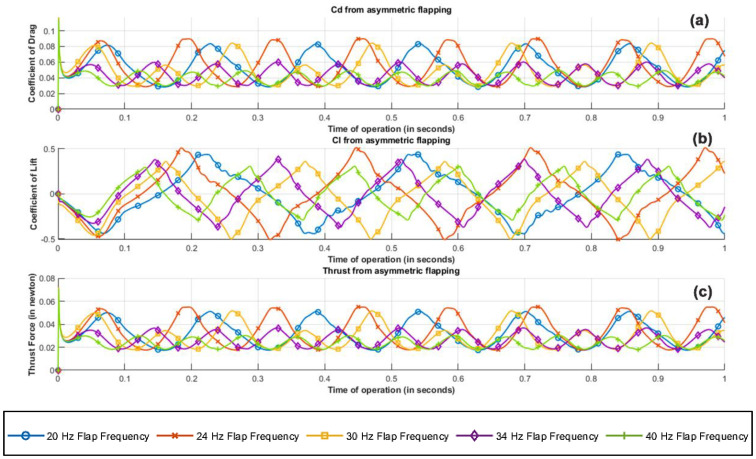
Averaged flow field metrics of left and right asymmetric flapping plate configurations; (**a**) coefficient of drag, (**b**) coefficient of lift, (**c**) thrust force along the positive x axis.

The resulting values for the left and the right wings are averaged to compute the net coefficient of lift, coefficient of drag, and the resultant thrust, as shown in Figure 21. The fluctuation in the simulated and computed parameters from 0 to 0.1 s is a relic of the emulation of gradual velocity ramping in the CFD simulation, similar to that of a wind tunnel blower, from 0 to 3.5 m/s. This gradual ramping tries to emulate the initial unstable inertial effects over the test body, synonymous to those induced over the body in actual experimental conditions until the flow is stabilized in the test section. However, for any meaningful interpretation of the obtained result, it is fair to neglect the logged results from 0 to 0.1 s.

Based on the results obtained from parametric study, a flapping frequency of 24 Hz seems ideal for the following reasons:

- The increase in the net coefficient of lift is more pronounced than the increase in drag by a factor of 22.7%.
- The power required to induce a flapping frequency of 24 Hz is relatively low compared to its higher-frequency counterparts, thus giving better output for 3 volts input.
- From the body’s vibrational standpoint, although explicit study hasn’t been performed to validate any claim, it is an engineering consideration that a high frequency requires faster mechanical motions, sometimes pushing the mechanical limit of microcomponents, resulting in vibrational and thermal management issues, leading to reduced life span.

## 5. Experimental Setup and Testing Results of Design Mechanism-II

**Figure 22:**
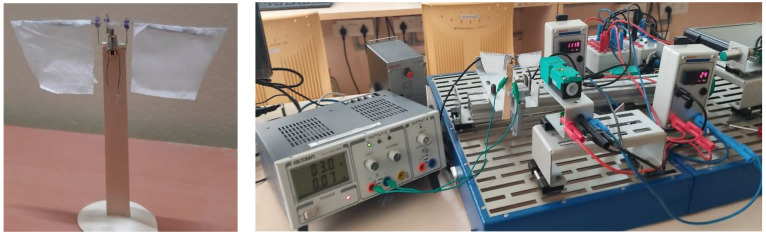
SC-SDL design II with Flapping Frequency Measuring Setup.

Fabrication of the proposed prototype, i.e., design mechanism-II, is carried out by hand, as a test bench model for the basic tests, is as shown in Figure 22. With the limited materials availability, the working prototype with necessary circuit connections is created and to actuate the wings, miniature brushless DC motors are installed. One of the crank-slider mechanisms has been accomplished in this flapping actuation design, as outlined above. The crank’s rotational motion is converted into a specified angular flapping motion at both the levers. The flapping action is enhanced by incorporating low friction and quick mechanical transmission into the design. The wing is firmly connected at the output link of the proposed model, which transmits the rotational motion of the crank to specific angular flapping motion. The design features low friction and rapid mechanical transmission, which are essential for improving flapping motion. It is observed that a flapping angle range between 25–35 degrees using this actuation system is achieved. The consistency of the actuator’s flapping is measured using a photoelectric sensor, and its flapping frequency is determined using a counter, as seen in Figure 22.

**Figure 23:**
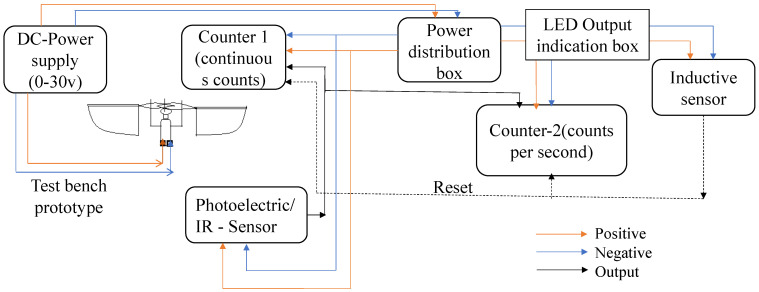
Schematic diagram of flapping frequency measuring setup.

Using an IR (infrared) sensor, the flapping frequency of the actuator can be determined, as shown in Figure 22, using a counter and tachometer in the circuit. There are many circuit components detailed in Figure 23, which illustrates the Flapping Frequency Measuring Setup via a schematic diagram. Using our prototype wing as a reference, a calibrated IR sensor is set to a sensing distance of 65 mm, as indicated in Figure 23. As the infrared sensor receives a DC voltage source of 24 v, and the DC-motor associated with the prototype receives 3 volts, it has successfully reported the number of flaps recognized by the sensor (i.e., 1118) at the counter-1. As seen in Figure 23, counter-2 is also configured for tachometer measurements to calculate the number of flaps per second. In addition, an inductive sensor is attached to refresh the value of both counters depending on the requirement through any metal part.

**Figure 24:**
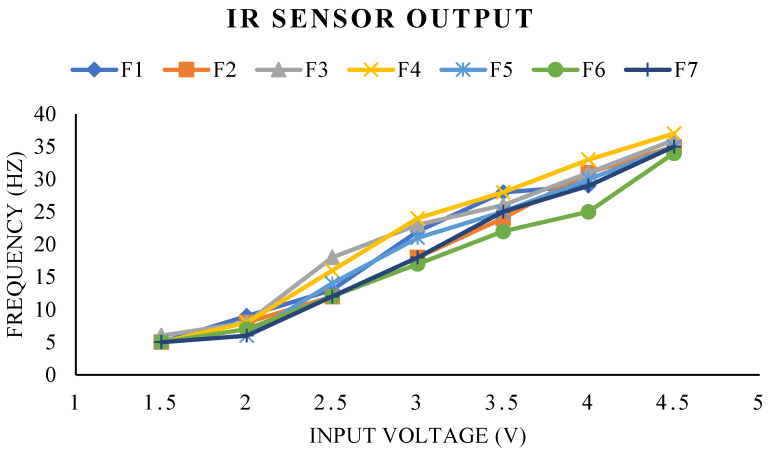
Variation in flapping frequency due to the variable input voltage of the motor.

As shown in Figure 24, the frequency readings from an IR sensor vary based on the motor’s input voltage. As the input voltage increases from 1.5 to 4.5 volts, the IR sensor’s flapping measurement exhibits a modest linear variation. Figure 24 illustrates that our proposed prototype can achieve a maximum frequency of 35 Hz at a maximum voltage of 4.5 v, here F1 to F7 are the several sets of measurements conducted to observe the differences in flapping frequency measurements. It is seen that the greatest fluctuation in flaps per second occurs between the 2.5 to 4 volts range of input.

**Figure 25:**
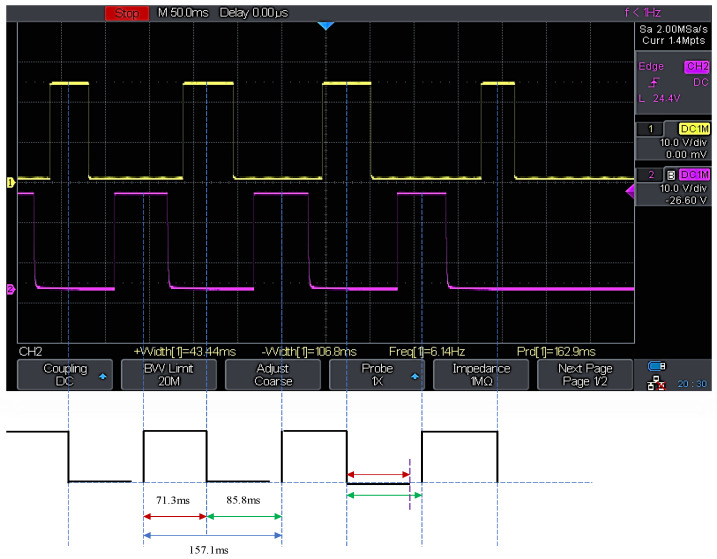
Digital CRO forward and reverse stroke time difference measurement.

Two IR sensors are positioned side-by-side, and the flapping wing is situated between both sensor wavelengths at a distance of 75 mm, such that the sensors can detect the forward stroke edge and return stroke edge. The time difference between forward and reverse strokes is determined using the CRO channels associated with each sensor. Figure 25 depicts the signals collected from the CRO, where the yellow signal shows the detection of the forward stroke edge, and the pink signal indicates the detection of the return stroke edge by the sensors. Figure 25 reveals that the forward stroke time space is 85.8 milliseconds, and the return stroke time space is 71.3 milliseconds, based on the time space between the yellow and pink signals. As the forward stroke requires more time than the return stroke, this confirms that the quick return mechanism in this system is functional. The above obtained signals are for a 6.14 Hz flapping frequency at an input voltage of 1.5 v.

**Figure 26:**
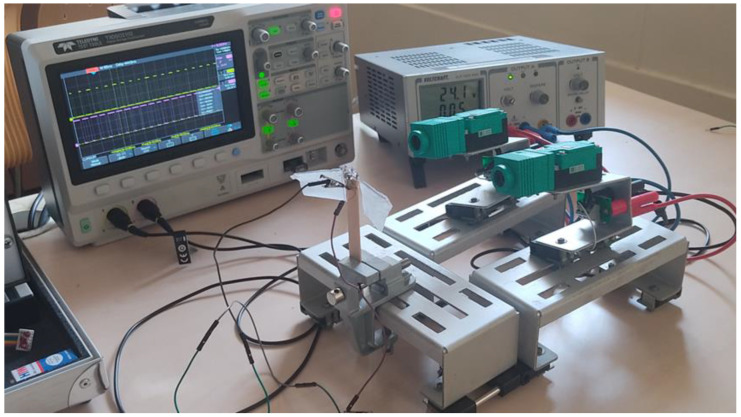
Phase lag between left- and right-wing measuring setup.

**Figure 27:**
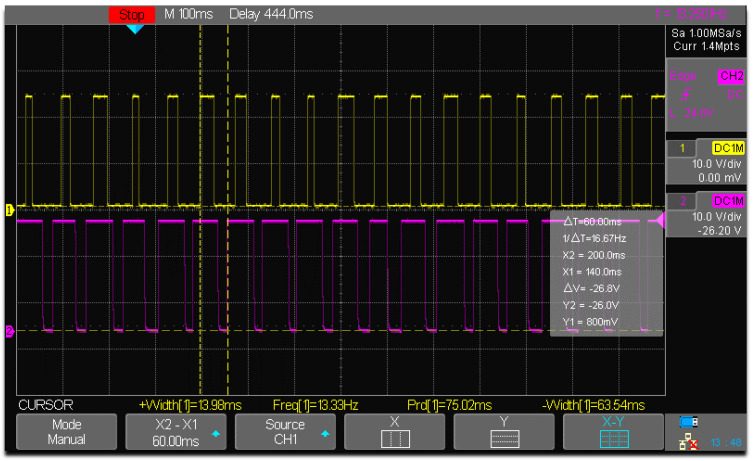
Phase lag between left and right wings.

As shown in simulations, structural design constraints caused a delay between the left- and right-wing positions during its flapping movements. i.e., approximately 2 degrees of angular position lag in one of its wings. In the proposed experimental setup using the prototype, it is attempted to evaluate the positional lag between the wing’s movements by mounting two IR sensors side-by-side, focused on each wing tip, as depicted in Figure 26**.** The CRO channels are connected to both sensors, as well as a 24 v DC input source that activates the sensors. As illustrated in Figure 27, the prototype is accelerated by an input voltage of 2.5 v, and able to acquire the flapping frequency of the wings measured at CRO as 13.33 Hz and 13.250 Hz. In the typical ‘clicking mechanism prototype’ of Y.W. Chin [40], the click happens when the stroke reaches a vital point. When a position lockout occurred in the crank slider mechanism, the study shows that a fast, rapid flap stroke (click) could raise wing-joint strains and splinter the wing at its joint interconnection. It was projected that the click mechanism will yield more kinetic energy in flapping than a resonant system, assumed for this specific work. It is seen that our mechanism is superior to clicking mechanisms in terms of flapping frequency actuation (as each individual wing is controllable), reduced weight, and low power consumption.

**Figure 28:**
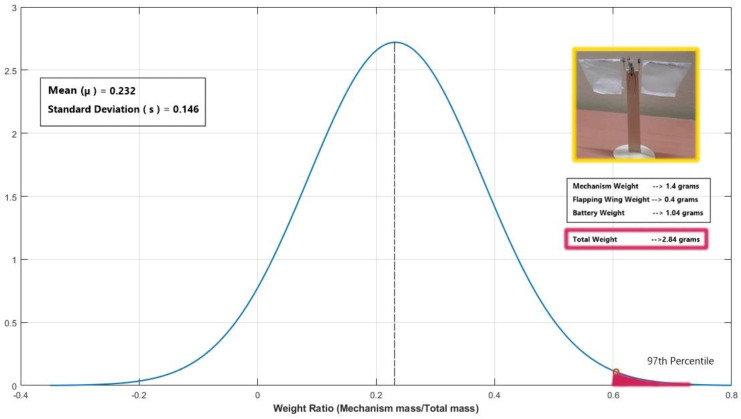
Normal Distribution comparison of the proposed design−II configuration’s metric against the existing flapping wing design weight ratio metric.

The overall weight of the prototype is 2.84 g (where weight of: motor = 0.54 g, battery = 1.04 g, two wings = 0.4 g, mechanism with only supporting structure = 0.86 g). For the proposed prototype design II configuration, the weight ratio calculation obtained is around 0.634, which safely falls in the 97th percentile (as shown in Figure 28) of all the nine best available weight ratios for micro flapping wing designs of a crank slider type mechanism from the previous literature [20], such as SF-3 prototype [41], Nguyen’s Beetle [42], Flapping robot (Lever crank mechanism) [43], Lung-Jieh Yang’s MAV [44], untethered 3.2 g FWMAV [45], Rotor-Bee [46], Evans Modified 3D printed Mechanism [47], FWR MAV [48], and Hassanalian’s FWMAV2 [49].

## 6. Future Work

Any compact flapping-win designs with the least size possible and involving interconnecting elements innovation is a difficult task. For completely flyable flapping-wing drone models, it is preferable to use a directly driven mechanism of rotary-actuated systems with a crank shaft or crank slider concepts [20,50,51]. One of the critical factors for comprehensive analysis of the kinematics and aerodynamics of any flapping wing body is to account for the flexibility in the wing structure and the associated three-dimensional effects of the ornithopter. From a physical standpoint, a net axial twist of the wing is experienced due to the center of pressure and shear center not coinciding in reality. A better prospect of extending the current study would be to understand the three-dimensional effects of the non-simplified geometry whilst accounting for wing flexibility, symmetric quick return, and wing twist. From noteworthy inferences in previous ornithopter works, it was well-validated that a hike in the dihedral angle will result in a net increase in the aerodynamic forces and thrust produced. Dynamic change of the dihedral angle pivoted at the trailing edge is a bioinspired spinoff, often found in near the fling mechanisms of some insects and birds. The incorporation of such dynamic change in the dihedral angle in response to the angle of attack and the freestream velocity will improve the existing mechanism and offer better chances of a stable and efficient design.

Finally, the current design suffers from a nonsymmetric return owing to the employed mechanism’s rotation. However, in some cases, this nonsymmetric flapping could also be advantageous. A very good starting point for future designs might incorporate symmetric flapping in addition to incorporating all the above-mentioned features and thus could be utilized in controls of a flapping-wing air vehicle through pointing the resultant force in the desired direction of flight.

## 7. Conclusions

This work outlines the construction and analysis of a biomimetic flapping actuation model consisting of a crank and sliding lever mechanism that represents the concept of SC-SDL. Two distinct SC-SDL designs are developed in CAD and analyzed using simulations, and comparisons are performed. Two-dimensional analysis of base ornithopter configuration using ANSYS FLUENT yielded insights about flapping aerodynamics and on the influence of varying flapping frequency on critical flow metrics regarding adequate lift and thrust produced. The proposed SC-SDL, dual-pivoted, sliding, single-crank mechanism (Design II) brings three significant improvements in comparison to its flapping-wing counterparts: it facilitates efficient power management and less mechanical complexity, coupled with a relatively streamlined aerodynamic interaction (L/D ratio of 4.721). The proposed Design II configuration safely falls in the 97th percentile of all the nine best available weight ratios for micro flapping wing designs from the literature. The current design renders a peak thrust-to-weight ratio of 21.148, validating its utility for sustained flight. For the current concept of SC-SDL configuration, a flapping frequency of 24 Hz proved to be effective with a peak lift generation coefficient of 0.293 whilst having minimal flow disturbances and wake interactions (peak Cd of just 0.0621). At various input voltages, structural design assessments of the flapping actuation mechanism determine the wing’s angular velocity, acceleration, and flapping frequency. The concept analysis is tested using experimental data gathered from the constructed prototype, such as the flapping-wing testbed. Separate infrared sensor measurement setups are created and analyzed for the testbed model to validate its flapping frequency. It has been noticed that there is a lag between both wing motions, confirming the asymmetric coordination of wing movement. Consequently, the revised SC-SDL model is adopted to minimize the lag. Based on obtained results and tests, it can be confirmed that the proposed SC-SDL mechanism is best suited for FWMAVs with future modifications of obtaining symmetric flapping capabilities with independent wing control concepts.

## Figures and Tables

**Table 1:** Mesh quality metric evaluation.

Total Number of Elements in the Domain			90,730	
Mesh Quality Metric	Minimum Value	Maximum Value	Overall Quality	Zone of Concern
Aspect Ratio (AR)	6.34	133	99.12% < AR = 7	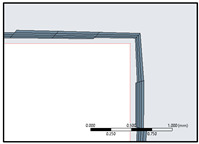
Orthogonal Quality (OQ)	0.171	0.956	98.76% > OQ = 0.85	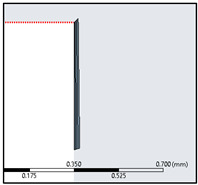
Skewness (SK)	0.0292	0.437	96.38% < 0.15 SK